# ﻿Molecular phylogenetics uncovers two new species in the genus *Phyllobates* (Anura, Dendrobatidae): the terrible frog gets two new sisters

**DOI:** 10.3897/zookeys.1212.126733

**Published:** 2024-09-16

**Authors:** Adolfo Amézquita, Fernando Vargas-Salinas, Iván Ramos, Pablo Palacios-Rodríguez, Erika Nathalia Salazar, Michelle Quiroz, Wilmar Bolívar, Diana M. Galindo-Uribe, Luis A. Mazariegos-H

**Affiliations:** 1 Laboratory of Biodiversity and Cloud Forests Conservation, Bioconservancy, Jardín, Colombia Laboratory of Biodiversity and Cloud Forests Conservation Jardín Colombia; 2 Grupo de Evolución, Ecología y Conservación (EECO), Programa de Biología, Facultad de Ciencias Básicas y Tecnologías, Universidad del Quindío, Armenia, Colombia Universidad del Quindío Armenia Colombia; 3 Facultad de Ciencias, Pontificia Universidad Javeriana, Bogotá, Colombia Pontificia Universidad Javeriana Bogotá Colombia; 4 Instituto de Investigación de Recursos Biológicos Alexander von Humboldt, Bogotá, Colombia Instituto de Investigación de Recursos Biológicos Alexander von Humboldt Bogotá Colombia; 5 Instituto Interdiciplinario de Investigación e Innovación, Vicerrectoría de Investigación y Postgrado, Universidad Autónoma de Chiriquí, Chiriquí, Panama Universidad Autónoma de Chiriquí Chiriquí Panama; 6 Grupo de Investigación en Ecología Animal, Departamento de Biología, Universidad del Valle, Cali, Colombia Universidad del Valle Cali Colombia

**Keywords:** Chocoan region, Colombia, hypertoxic frogs, molecular phylogenetics, *Phyllobates*, Chocó biogeográfico, Colombia, filogenética molecular, *Phyllobates*, ranas hipertóxicas

## Abstract

True poison-dart frogs (*Phyllobates*, Dendrobatidae) evolved the ability to secrete batrachotoxins, the most powerful alkaloids known to date. The genus comprises five species whose systematics, at first glance, appeared clear. The most derived clade would include two Colombian species (*P.terribilis* and *P.bicolor*) with the highest toxicity, the largest body size, and predominantly yellow body colouration. The other three species (*P.aurotaenia*, *P.vittatus*, and *P.lugubris*) are less toxic on average, have smaller size, and are predominantly black with bright dorsolateral stripes. Recent research has revealed the existence of two major lineages among the three Colombian species. The northern lineage appears to result from a complex evolutionary history, including perhaps introgression among yellow and black taxa. The southern lineage instead revealed the existence of new clades closely related to *P.terribilis*, black and yellow, that arguably deserve their recognition as new species. Here, available evidence is combined to support the erection of southern populations of *P.aurotaenia* as a new highly toxic species, sister to *P.terribilis*, and much closer to it than to any other yellow or black-bodied species, *Phyllobatessamperi***sp. nov.** Their common ancestor is sister to an additional yellow species, which we also describe here as *Phyllobatesbezosi***sp. nov.** Both new species can be externally diagnosed using colouration. Our previous and current analyses also suggest the existence of additional taxa and corroborate multiple transitions in colouration across these hypertoxic taxa.

## Abstract (es)

Las verdaderas ranas de dardo venenoso (género *Phyllobates*) evolucionaron la capacidad de secretar batracotoxinas, los alcaloides más poderosos que se conocen hasta hoy. El género comprende cinco especies, cuya taxonomía parecía clara a primera vista. El clado más derivado incluye dos especies colombianas (*P.terribilis* and *P.bicolor*) con la mayor toxicidad, mayor tamaño y coloración predominantemente amarilla. Las otras tres especies (*P.aurotaenia*, *P.vittatus* and *P.lugubris*) son en general menos tóxicas, menores en tamaño y predominantemente negras con conspicuas manchas dorsolaterales.Una reciente investigación confirmó la existencia dos linajes mayores entre las tres especies colombianas. El del norte parece ser el producto de una historia evolutiva compleja, incluyendo quizás introgresión entre especies amarillas y negras. El del sur reveló en cambio la existencia de nuevos clados filogenéticamente cercanos a *P.terribilis*, uno negro y dos amarillos, que requieren reconocimiento formal como nuevas especies. Esta investigación combina la evidencia disponible para apoyar el reconocimiento de las poblaciones del sur de *P.aurotaenia* como una nueva especie negra, hermana de *P.terribilis*, y mucho más cercana a ella que a las otras especies negras o amarillas, *Phyllobatessamperi***sp. nov.** Su ancestro común es a su vez el grupo hermano de una especie adicional, amarilla, que también describimos como *Phyllobatesbezosi***sp. nov.** Las dos nuevas especies pueden ser externamente reconocidas por la coloración. Nuestros análisis previos y actuales también sugieren la existencia de otras especies no descritas y corroboran la existencia de múltiples transiciones de coloración en la evolución de las especies hipertóxicas.

## ﻿Introduction

True poison dart frogs (*Phyllobates*, Dendrobatidae) were discovered by Chocó indigenous people in western Colombia and used to poison blowgun darts by rubbing them on the frogs’ backs (Cochrane 1825; Posada-Arango 1869; Saffray 1873; Wassén et al. 1935; Wassén 1957). The reported tradition called the attention of later researchers, who isolated the cocktail of toxins secreted by the frog skins (Märki and Witkop 1963; Daly et al. 1965) and demonstrated the presence of two toxic and potent alkaloids: batrachotoxin and homobatrachotoxin (Tokuyama et al. 1968, 1969). A new species was later formally described and found so toxic that Myers et al. (1978) named it *Phyllobatesterribilis*: the terrible leaf walker. The authors also proposed to restrict the name *Phyllobates* to a small species group, based on their unique ability to secrete batrachotoxins. After five decades of intensive research on the chemical ecology of cutaneous frog toxins (e.g. Daly et al. 2005; Saporito et al. 2012; Coleman and Cannatella 2023), the few *Phyllobates* species remain the only amphibians known to secrete batrachotoxins (Grant et al. 2006), which are among the most powerful neurotoxins known to science.

The genus *Phyllobates* sensu Myers et al. (1978) comprises five species, three distributed throughout the Western Andean and Pacific forests of Colombia, and two in the lowland Caribbean and Pacific versants of Costa Rica and Panamá. Only the Colombian species (*P.terribilis*, *P.aurotaenia*, and *P.bicolor*) were reportedly used to poison darts (Silverstone 1976; Myers et al. 1978). Studies on the systematics of *Phyllobates* (Maxson and Myers 1985; Widmer et al. 2000; Márquez et al. 2012), or dendrobatid frogs in general (Vences et al. 2003; Grant et al. 2006, 2017; Santos et al. 2009), have consistently confirmed the monophyly of the genus, as well as the reciprocal monophyly between two main subclades: one including the central American species (*P.vittatus* and *P.lugubris*) and another one grouping the Colombian species (*P.aurotaenia*, *P.bicolor*, and *P.terribilis*).

The evolutionary relationships between the three Colombian species appeared clear at first glance (Maxson and Myers 1985): *P.terribilis* was considered the sister species of *P.bicolor*, and their common ancestor the sister taxon of *P.aurotaenia*. This simple arrangement was supported by an allegedly derived and mostly uniform yellow dorsal colouration in *P.terribilis* and *P.bicolor*; in contrast, the dorsum was essentially black with two yellow, orange, or green dorsolateral lines in the basal *P.aurotaenia*, and in the more basal mesoamerican species. Other presumably derived conditions for the yellow species are larger body size and higher content of batrachotoxins in their skins. Initial mtDNA analyses further supported this phylogenetic hypothesis (Widmer et al. 2000).

It was suggested very early that *P.aurotaenia* may consist of two clades: the narrow-banded and broad-banded forms, differing in colouration and, perhaps, altitudinal distribution (Silverstone 1976). Other studies rendered mixed phylogenetic results for *P.aurotaenia* as a whole: the species was grouped with *P.terribilis* (Grant et al. 2006), *P.bicolor* (Santos et al. 2009), or both (Grant et al. 2017), although the latter arrangement was based on very few samples almost exclusively obtained from the pet trade. More recent and exhaustive studies supported the possibility of broad-banded forms resulting from introgression with *P.bicolor* (Márquez et al. 2020; González-Santoro et al. 2023). Altogether, the accumulated evidence questions the identity and the monophyly of the lineages currently assigned to *P.aurotaenia*.

In the course of field investigations on the evolution of hypertoxicity in poison frogs, we noticed unexpected genetic and colouration traits in a population currently assigned to *P.aurotaenia*. Moreover, we acknowledged the existence of another distinctive form, predominantly yellow in colouration, which has not been genotyped before. Both observations led us to question the taxonomic status of the involved lineages. A more recent analysis of gene flow among the Colombian species and populations of *Phyllobates* (Márquez et al. 2020), confirmed a major lineage division among the Colombian taxa, one to the north and one to the south, as well as the paraphyletic nature of some of them.

To the north, conspicuous geographic variation in colouration (Márquez et al. 2020) and advertisement calls (González-Santoro et al. 2023) appear to reveal an intricate history of gene flow and perhaps introgression among at least two taxa, currently assigned to *P.aurotaenia* and *P.bicolor*. To the south, two new clades appeared in addition to *P.terribilis*. We aim here to add evidence and improve the coherence between the taxonomic arrangement and the underlying evolutionary processes, by erecting two new species of the southern lineage, one predominantly black and one predominantly yellow, and both within the clade of Colombian hypertoxic frogs of the genus *Phyllobates*.

## ﻿Materials and methods

### ﻿Study area

The specimens used in this study were collected during fieldwork conducted between 2009 and 2022 in localities of the Colombian Chocó bioregion, where *Phyllobates* species occur. Specimens of the black clade were collected around Buenaventura, Magüipi, Chucheros, and San Cipriano, all within the Departamento del Valle (Colombia). Specimens of the yellow clade were collected around the Garrapatas river, near the border between Departamento del Chocó and Departamento del Valle del Cauca. We list further details in species descriptions.

After capture, most specimens were photographed and measured (snout-to-vent length, SVL); some were then released at the site of capture. A number were transported to the laboratory, euthanised using topical lidocaine, fixed in 10% formalin, and stored in 70% ethanol, to be deposited at the Amphibian Collection of the C.J. Marinkelle Museum of Natural History at Universidad de los Andes (**ANDES-A**), Bogotá, the Amphibians and Reptiles Collection at the Universidad del Valle (**UV-C**). Further specimens were deposited at the Herpetological Museum, *Universidad de Antioquia* (MHUA), Medellín, Colombia. Before fixation, fresh liver or muscle samples were collected for DNA analysis and stored in 95% ethanol.

### ﻿Phylogenetic analyses

To reproduce the phylogenetic hypothesis on the studied taxa (Márquez et al. 2020), we built a molecular matrix using a set of mtDNA sequences downloaded from GenBank for 98 individuals (Márquez et al. 2020) and newly obtained for five additional ones. The use of mtDNA alone follows a much more detailed approach in Márquez et al. (2020), that focused on RNA transcripts from the whole genome, rendering 32,216 contigs used for the coalescent phylogenetic analyses. The resulting phylogenetic tree was found to be largely concordant with another tree estimated using only mtDNA (Márquez et al. 2020).

Our matrix thus included three mitochondrial loci (16S rRNA, COI, and Cytb) using primers 16Sar and 16Sbr (Palumbi et al. 1991), dgLCO1490 and dgHCO2198 (Meyer et al. 2005), and CytbDen3-L and CytbDen1-H (Santos and Cannatella 2011), respectively. To obtain sequences from the five additional individuals of the yellow clade (Garrapatas River clade), we extracted DNA using the Qiagen DNeasy Blood and Tissue kits, following standard manufacturer protocols. The new sequences were deposited in GenBank (accession numbers in Suppl. material 1).

Homologous sequences of each region were aligned using MAFFT under default parameters (Katoh and Standley 2013). The best partition scheme and the model of DNA evolution were estimated using ModelFinder (Kalyaanamoorthy et al. 2017). To infer a maximum likelihood tree and the nodal support, we used the ultrafast bootstrap method on 5000 pseudoreplicates as implemented in IQTREE 2.3.4 (Hoang et al. 2018; Minh et al. 2020). All other parameters were left at their default values.

To estimate the degree of genetic divergence among the focal Colombian lineages, we used a subset of 42 (16S rRNA), or 43 (COI and Cytb) sequences. We chose these individuals because we had a complete set of DNA sequences for all three genes, with one exception (Suppl. material 1). We excluded individuals previously reported from the pet trade because they lack information on phenotype and locality. We also excluded individuals of *P.aurotaenia* from the upper San Juan river (broad band forms in Silverstone 1976) given their dubious taxonomic affinity and the probable hybrid origin (Márquez et al. 2020; González-Santoro et al. 2023), which is beyond the scope of this paper. To depict among-species genetic distances for each gene, we estimated uncorrected *P* genetic distances using MEGA (Kumar et al. 2016). To visualise among-species distances in two dimensions, we used multidimensional scaling on the distance matrix and built two-dimensional plots, one per mitochondrial locus. To test for the presence of a distance gap we further estimated among-individual genetic distances, both within- and between the species, and depicted graphically those focusing on the species of the southern lineage, including the two new species we describe herein.

### ﻿Morphological data

Using a digital calliper, we took the following measurements of the preserved adults: length from snout to vent (**SVL**), femur length from flexed knee to groin (**FL**), tibia length between heel and outer surface of flexed knee (**TL**), greatest body width, measured under armpits (**GBW**), head width between angles of jaws (**HW**), head length from tip of snout to angle of jaw (sagittal; **HL**), horizontal eye length (**EL**), internarinal distance (**IN**), interorbital distance (**IOD**); distance from centre of naris to anterior edge of the eye (**NED**), horizontal diameter of the tympanum (**HDT**), left-hand length from palmar tubercle to longest (third) finger (**HaL**), and left foot length from inner metatarsal tubercle to longest (fourth) finger (**FoL**). For tadpoles, we measured the body length (**BL**) and tail length (**TaL**) following Altig and McDiarmid (1999), which we added to get the total length (**ToL**).

Two of the captured males of the black clade were found carrying four back-riding tadpoles; the eight tadpoles were euthanised in a lidocaine solution upon capture, preserved in 95% ethanol, and stored in 70% ethanol. Moreover, a pair of frogs produced a fertile egg clutch while housed in the laboratory; one captive-born tadpole was raised until Gosner’s developmental stage 31 (Gosner 1960), and then preserved as detailed above. One pair of the yellow clade bred twice under laboratory conditions. The female laid nine eggs in each case. Eggs and tadpoles were raised in separate containers. Eight individuals were kept after metamorphosis and two tadpoles were preserved under the conditions described above.

### ﻿Call recording

We recorded at least seven spontaneous advertisement calls for each of ten males of the black clade at three localities: Piangüita (*n* = 8 males, including the holotype), Bahía Málaga (*n* = 1) and Bajo Calima (*n* = 1), all in the vicinities of Buenaventura city, Colombia. Calls were recorded using a Marantz PMD660 digital recorder, at 16-bit resolution and 44.1 kHz sampling rate, and a unidirectional microphone (AKG D-190-E, Shure BG4.1, or Sennheiser ME-62/K6), positioned at a distance of 0.5–2.0 m in front of the male. We also recorded two and seven calls of two males of the yellow clade using the same equipment and parameters under laboratory conditions. Body temperature was measured after recording with an infrared thermometer positioned at ~ 10 cm of male dorsum.

To allow for proper across-species comparisons in the future, we recognise a homologous sound unit as the sound produced by a single thoracic compression (Erdtmann and Amézquita 2009). We used the R package seewave (Sueur et al. 2008) to measure five temporal parameters on oscillograms and two spectral parameters on spectrograms, the latter estimated with a Fast Fourier Transformation window of 1024 points under the Blackman algorithm. To avoid pseudoreplication, the average value of the recorded calls for each frog represents the unit for statistical descriptions.

## ﻿Results

### ﻿Phylogenetic relationships

**Figure 1.**
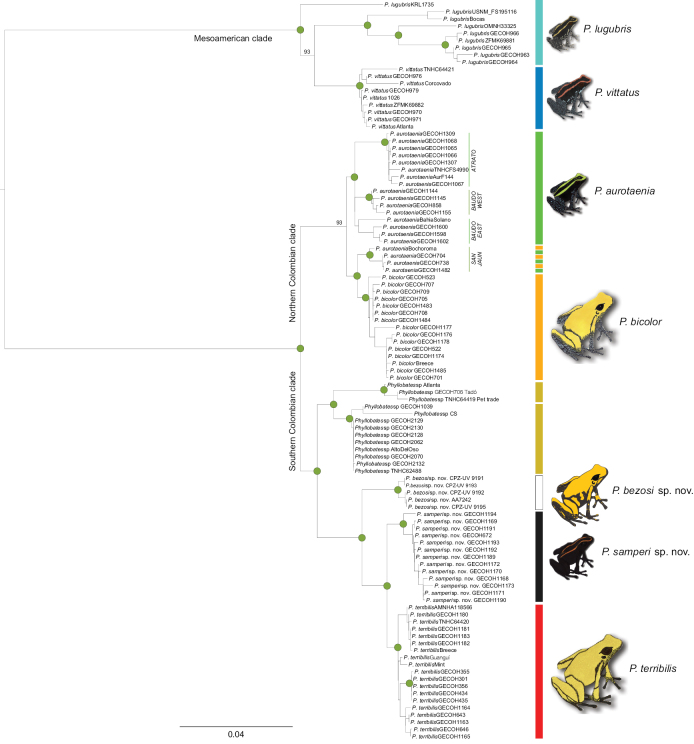
Recovered relationship between Colombian species and clades of *Phyllobates* and proposed taxonomic arrangement in the light of current evidence. Green dots denote nodal support (UFBoot2) of at least 95%. The individuals outlined by two-coloured bars remain without a name, because they represent the probable result of introgression or hybridisation (Márquez et al. 2020). See Materials and methods for further details on the phylogenetic analysis and Suppl. material 1 for localities and codes of specimens. Frog icon colouration is approximate and should not be used to obtain diagnostic traits. See Fig. 4 for a colour diagnosis of the Colombian species.

The final dataset consisted of 1926 mtDNA nucleotides (16S: 568 bp; COI: 658 bp; Cytb: 700 bp) aligned for 103 individuals and including 425 parsimony-informative and 68 singleton sites. The mtDNA analysis essentially reproduced the previously published phylogenetic hypothesis either based on RNA extracts of the whole genome or in mtDNA alone (Márquez et al. 2020): two clades formed at the root of *Phyllobates*, one containing the two Central American species, and another one containing the Colombian species (Fig. 1). Within the Colombian clade, two clades were recovered as well: one to the north and one to the south in the Pacific lowlands. Within the latter, the black clade (*P.aurotaenia* South sensu Márquez et al. 2020), was reciprocally monophyletic to *P.terribilis* rather than to other populations of *P.aurotaenia*. In turn, the newly discovered yellow clade was sister to the common ancestor of the former two. In addition to this, an already reported clade (*P.bicolor* South in Márquez et al. 2020) was sister to the common ancestor of the former three. Because no individuals were available of the latter clade, we refrain from formally describing it as a new species.

**Figure 2.**
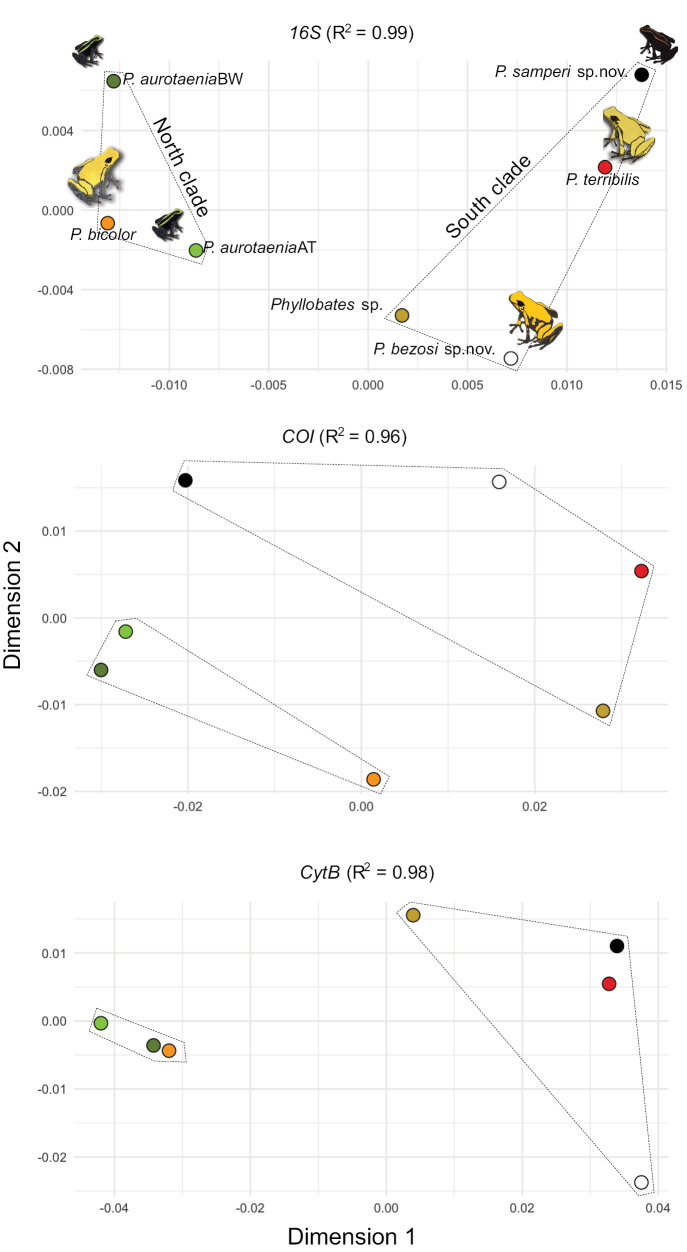
Two-dimensional representation of genetic (16S, COI, and Cytb) uncorrected P-distances between clades of Colombian *Phyllobates*, including the two new species described herein. Two distinctive forms of *P.aurotaenia* are shown: the Baudo West and Atrato River forms in Márquez et al. (2020). The axes are unitless and derived from Classic Multidimensional Scaling of pairwise genetic distances, which accounted for 96–99% (see R^2^) of the variation in the original genetic distances. Species names, dot colours, and polygon identities are presented on the top plot and should be read in the other two plots. See Fig. 1 for the recovered phylogenetic relationships of the presented clades.

**Figure 3.**
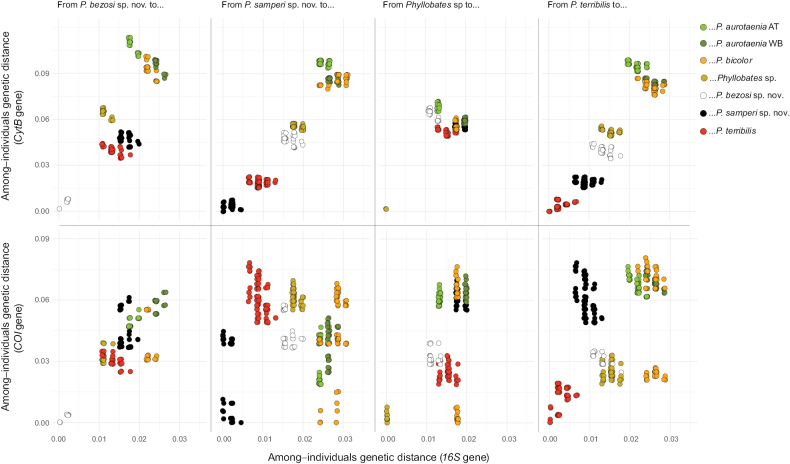
Two-dimensional distribution of genetic (16S, COI, and Cytb) distances between individuals of the same or different operational taxa addressed in this research. To read each panel, start from the panel title and then use the colour legend to identify the kind of comparison depicted by each uniquely coloured set of data points. To facilitate reading, only comparisons from species in the southern lineage are shown, which includes the newly described species.

The pairwise genetic distances between each of the new species and all other species further corroborated their status as independent lineages (Table 1). Overall, the black clade was indeed much closer to *P.terribilis* than to any form of *P.aurotaenia*. The original among-species distances were very precisely described by the multidimensional scaling analyses: the R^2^ values were between 96 and 99% (Fig. 2). In the corresponding plots (Fig. 2), the distances between the two new species and the previously recognised taxa were fairly similar to the genetic distances among the latter. The gap analyses further corroborated this pattern. There was no overlap between the within-species and among-species genetic distances when plotted in the two-dimensional spaces created by pairs of genes (Fig. 3).

**Table 1.** Genetic distances. Uncorrected p-genetic (16S, COI, and Cytb) distances among species and operational taxa of *Phyllobates* addressed in this study. Standard errors (95%) were estimated from 999 bootstraps and are presented above diagonal.

Clade	auAt	auBw	bezo	bico	sp	samp	terr
	16S						
*P.aurotaenia* At		0.005	0.006	0.004	0.005	0.007	0.007
*P.aurotaenia* Bw	0.011		0.007	0.004	0.007	0.008	0.007
*P.bezosi* sp. nov.	0.018	0.025		0.007	0.005	0.006	0.005
*P.bicolor*	0.009	0.010	0.023		0.006	0.008	0.007
*P.* sp. (*bicolor* South)	0.013	0.020	0.012	0.018		0.006	0.006
*P.samperi* sp. nov.	0.025	0.027	0.017	0.028	0.018		0.004
*P.terribilis*	0.022	0.026	0.014	0.026	0.015	0.009	
	COI						
*P.aurotaenia* At		0.006	0.009	0.006	0.010	0.006	0.010
*P.aurotaenia* Bw	0.020		0.010	0.007	0.010	0.007	0.010
*P.bezosi* sp. nov.	0.047	0.055		0.007	0.007	0.008	0.007
*P.bicolor*	0.039	0.044	0.042		0.005	0.006	0.007
*P.* sp. (*bicolor* South)	0.057	0.060	0.031	0.033		0.009	0.006
*P.samperi* sp. nov.	0.032	0.037	0.044	0.042	0.058		0.009
*P.terribilis*	0.063	0.064	0.029	0.045	0.024	0.058	
	Cytb						
*P.aurotaenia* At		0.005	0.011	0.006	0.009	0.010	0.010
*P.aurotaenia* Bw	0.016		0.011	0.005	0.009	0.010	0.010
*P.bezosi* sp. nov.	0.085	0.076		0.010	0.009	0.008	0.007
*P.bicolor*	0.026	0.016	0.074		0.008	0.010	0.010
*P.* sp. (*bicolor* South)	0.058	0.051	0.054	0.048		0.008	0.008
*P.samperi* sp. nov.	0.078	0.071	0.041	0.071	0.049		0.005
*P.terribilis*	0.076	0.069	0.035	0.068	0.045	0.018	

### ﻿Systematics

#### ﻿ Phyllobates samperi

Taxon classificationAnimaliaAnuraDendrobatidae

sp. nov.

E7A7013D-5DA4-5550-A9ED-974B77792E91

https://zoobank.org/C36AEDD2-5E4C-4149-B579-7B7DB6C1A144

Figs 4 , 5 , 6 , 7

P. aurotaenia : Silverstone 1976: 23; USNM-145105, USNM-145106.
P. aurotaenia : Márquez et al. 2020: 3703–3714, figs 1–5; South.

##### Type material.

**Figure 4.**
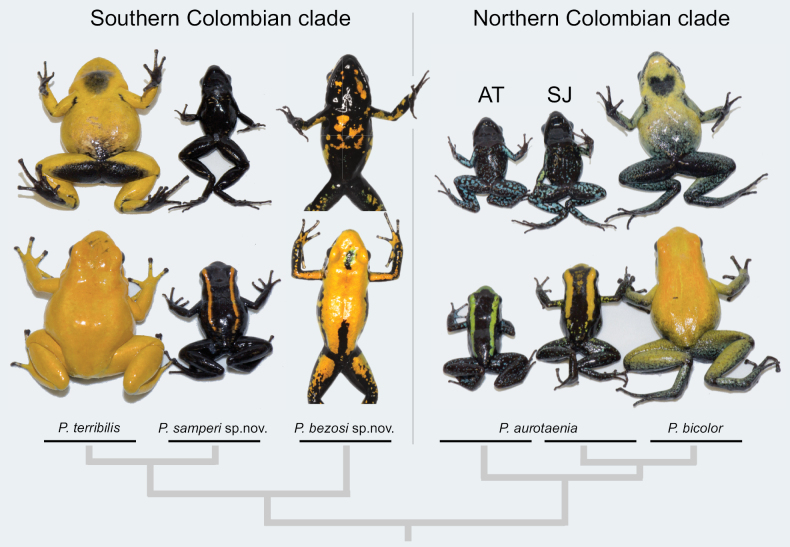
Dorsal and ventral view of *Phyllobates* species in Colombia, including the narrow-banded (*P.aurotaenia* AT, Atrato in Fig. 1) and broad-banded (*P.aurotaenia* SJ, San Juan in Fig. 1) morphs of *P.aurotaenia*. The tree topology follows the results depicted in Fig. 1.

***Holotype*** • Adult male. Field original label: 16B. Museum ID: ANDES-A 3673. Type locality in Colombia, Valle del Cauca, municipality of Buenaventura, 3.84°, -77.20°, 10 m elevation, in the village of Piangüita, within a patch of moderately disturbed rainforest, amidst the leaf litter, July 2020. Collected by Adolfo Amézquita. Advertisement calls of this male (Fig. 4) were recorded immediately before the capture. Measurements of this individual are in Table 2. ***Paratopotypes*** • Twelve males, two females and three non-sexed individuals. ANDES-A 3666–72, and UVC 15332 from the type locality, collected by Adolfo Amézquita and Fernando Vargas-Salinas, in July 2010 (ANDES 3666–72), and previously by Fernando Castro-Herrera in April 2005 (UVC-H 15332). ***Paratypes*** • Seven non-sexed individuals. ANDES-A 3675–78, 3680–81 from Colombia, Valle del Cauca, municipality of Buenaventura, Magüipi. ANDES-A 3679 from Chucheros and ANDES-A 3674 from the vicinities of San Cipriano, same municipality in both cases, collected in July–August of 2011 by Fernando Vargas-Salinas. UVC 7878–79, collected in Bajo Calima, Buenaventura in March of 1985, and UVC 8849 collected in Bahía Málaga, Buenaventura in March of 1985 by Fernando Castro-Herrera. Measurements of these individuals are presented in Table 2. MHUA-A 13245 and MHUA-A 13273 from Colombia, Valle del Cauca, Buenaventura, Magüipi, collected by Juan Manuel Daza.

**Table 2.** Morphometry. Body measurements and sex of collected individuals of the new species that are described herein. HT denotes holotype. See Materials and methods for abbreviations.

Species	ID	Sex	SVL	FL	TL	GBW	HW	HL	EL	IN	IOD	NED	HDT	HaL	FoL
*P.samperi* sp. nov.	ANDES-A 3673 (HT)	M	24.0	11.8	13.7	9.0	8.3	9.3	3.3	3.2	5.1	2.4	2.8	6.2	10.4
*P.samperi* sp. nov.	UVC-H 7878	F	27.0	11.2	13.6	8.2	8.6	9.2	3.4	3.6	5.8	3.2	1.8	6.5	12.5
*P.samperi* sp. nov.	UVC-H 7879	M	21.7	9.0	11.5	7.7	6.5	7.6	3.5	2.4	5.9	2.4	1.4	5.7	9.7
*P.samperi* sp. nov.	UVC-H 8849	–	23.1	9.5	11.5	7.2	7.4	8.1	3.3	3.7	5.0	2.4	1.1	6.1	10.9
*P.samperi* sp. nov.	UVC-H 15332	M	21.5	8.0	11.6	6.9	6.4	8.5	2.7	3.2	4.2	2.5	1.9	5.5	9.4
*P.samperi* sp. nov.	ANDES-A 3679	M	26.5	10.6	12.7	7.4	8.9	8.0	3.1	2.7	5.8	2.7	1.3	5.8	9.5
*P.samperi* sp. nov.	ANDES-A 3678	–	25.9	9.3	12.8	8.1	7.8	8.0	2.7	2.7	4.4	2.5	1.2	5.6	11.1
*P.samperi* sp. nov.	ANDES-A 3677	–	26.1	10.8	13.9	8.9	7.7	9.2	3.1	2.8	5.7	2.6	1.1	6.1	11.0
*P.samperi* sp. nov.	ANDES-A 3676	M	25.6	10.6	12.6	9.2	7.7	8.0	2.5	3.0	4.7	2.5	1.2	6.2	11.0
*P.samperi* sp. nov.	ANDES-A 3675	M	25.4	9.2	11.2	9.3	8.9	9.3	3.9	2.8	4.6	2.6	1.2	5.8	10.7
*P.samperi* sp. nov.	ANDES-A 3674	M	25.7	9.2	12.8	9.3	8.0	8.0	3.9	3.0	5.7	2.5	1.1	5.7	11.1
*P.samperi* sp. nov.	ANDES-A 3666	M	24.1	12.3	12.4	7.7	7.5	9.2	3.6	2.4	5.7	2.0	2.8	7.1	12.4
*P.samperi* sp. nov.	ANDES-A 3667	M	25.6	10.2	13.8	9.8	8.6	9.9	3.8	3.7	5.9	2.5	2.8	6.4	11.2
*P.samperi* sp. nov.	ANDES-A 3668	M	25.9	11.6	12.4	8.7	7.6	9.5	3.5	3.4	6.4	2.4	2.8	6.3	10.0
*P.samperi* sp. nov.	ANDES-A 3669	M	25.0	11.2	14.4	7.2	7.6	8.5	3.3	3.7	5.8	3.6	2.7	6.5	11.0
*P.samperi* sp. nov.	ANDES-A 3670	F	27.0	10.7	13.7	8.6	8.2	9.0	3.2	3.0	5.5	2.5	2.9	6.4	11.9
*P.samperi* sp. nov.	ANDES-A 3671	M	24.5	11.3	13.6	7.1	7.2	8.7	3.3	4.3	5.4	2.4	2.8	6.1	11.8
*P.samperi* sp. nov.	ANDES-A 3672	–	24.5	11.7	12.3	7.1	7.4	9.9	3.3	3.0	5.2	2.6	2.8	5.8	11.9
	Mean		25.0	10.5	12.8	8.2	7.8	8.8	3.3	3.1	5.4	2.6	2.0	6.1	11.0
	SD		1.6	1.2	1.0	0.9	0.7	0.7	0.4	0.5	0.6	0.3	0.8	0.4	0.9
*P.bezosi* sp. nov.	AA_7242	–	32.9	15.5	17.0	13.0	11.5	8.9	4.8	3.3	4.1	3.2	3.1	10.4	16.2
*P.bezosi* sp. nov.	AA_7243	–	32.9	16.3	17.0	13.4	11.2	12.1	4.8	3.6	4.3	3.6	3.4	10.2	16.7
*P.bezosi* sp. nov.	AA_7244	–	35.3	15.4	16.7	12.2	11.6	10.5	4.5	3.8	4.9	4.7	3.2	9.4	17.4
*P.bezosi* sp. nov.	AA_7245	–	30.9	14.0	16.6	12.0	12.9	7.5	4.6	4.3	7.5	4.1	2.8	9.1	14.2
*P.bezosi* sp. nov.	CPZ-UV 9191 (HT)	M	32.6	16.2	17.6	10.9	10.8	9.4	4.8	4.5	3.4	3.1	2.6	8.9	15.2
*P.bezosi* sp. nov.	CPZ-UV 9192	M	34.2	16.4	18.5	11.1	11.6	10.5	5.2	5.3	3.3	3.6	2.6	10.2	16.4
*P.bezosi* sp. nov.	CPZ-UV 9193	M	35.0	17.1	18.2	12.2	12.3	10.5	5.0	5.2	3.4	3.1	2.6	9.2	16.9
*P.bezosi* sp. nov.	CPZ-UV 9194	M	32.3	16.0	17.6	12.0	10.9	9.3	5.2	5.2	3.5	3.1	2.7	8.9	14.7
*P.bezosi* sp. nov.	CPZ-UV 9195	F	36.1	17.6	19.7	13.1	12.1	10.8	5.4	5.7	4.3	3.6	3.0	10.1	17.0
	Mean		33.6	16.0	17.6	12.2	11.7	9.9	4.9	4.5	4.3	3.6	2.9	9.6	16.1
	SD		1.7	1.0	1.0	0.9	0.7	1.3	0.3	0.9	1.3	0.5	0.3	0.6	1.1

##### Diagnosis.

*Phyllobatessamperi* sp. nov. is a small to medium-sized dendrobatid with an adult SVL of 21–27 mm (24.5 ± 1.6 mm, mean ± SD, *n* = 17). It is mostly pitch-black, with a narrow golden yellow/orange complete dorsolateral stripe (sensu Grant et al. 2006) extending from the snout, along the outer margin of the upper eyelid, to the dorsum near the dorsal base of the thigh. The ends of the stripe do not meet at the urostyle. Some individuals exhibit a few yellow/orange specks on the forearms, legs and/or venter. We assign this species to *Phyllobates* based on other studies with more extensive outgroup sampling, including molecular (Santos et al. 2009) and combined (with morphological traits) phylogenetic analyses (Fig. 1; Grant et al. 2006, 2017; Márquez et al. 2020). We also use the following combination of traits: finger I longer than finger II, maxillary teeth present, call of the trill type (Myers and Daly 1976; Myers et al. 1978), and the ability to secrete batrachotoxin (T. Escovar, M.C. González, and A. Amézquita, pers. obs.).

##### Comparisons.

*Phyllobatessamperi* sp. nov. can be distinguished from other members of its genus as follows: colouration and size easily separate *P.samperi* sp. nov. from *P.terribilis*, *P.bicolor*, and *P.bezosi* sp. nov. (Fig. 4). The latter three have plain or predominant yellow, orange, blueish white, or mint-green dorsal colouration, and are much larger than *P.samperi* sp. nov. The froglets of the three yellow species exhibit a bright dorsolateral stripe shortly after metamorphosis, but the stripe is much wider than the one of *P.samperi* sp. nov. and turns into plain colouration early in development when froglets are ~ 20 mm in SVL. The absence or near absence of blue colouration distinguishes *P.samperi* sp. nov. from colour morphs of the putative *P.aurotaenia*, which exhibit abundant blue or silver specks or marbling in the venter and limbs. The limbs and venter are uniformly black in most specimens of *P.samperi*, with a few, very small and sparse orange or blueish specks occurring in some individuals (Fig. 4). The absence or near absence of blue can also be used to separate *P.samperi* sp. nov. from *P.bicolor*, which exhibits blue/green pigmentation on the hind limbs, although this trait has been argued to be absent in some populations of this species (Lötters et al. 1997 but see Márquez et al. 2012). Furthermore, *P.samperi* sp. nov. can be distinguished from the Central American species *P.vittatus* and *P.lugubris* by the absence of ventrolateral stripes and ventral marbling in *P.samperi* sp. nov.

##### Description of the holotype.

**Figure 5.**
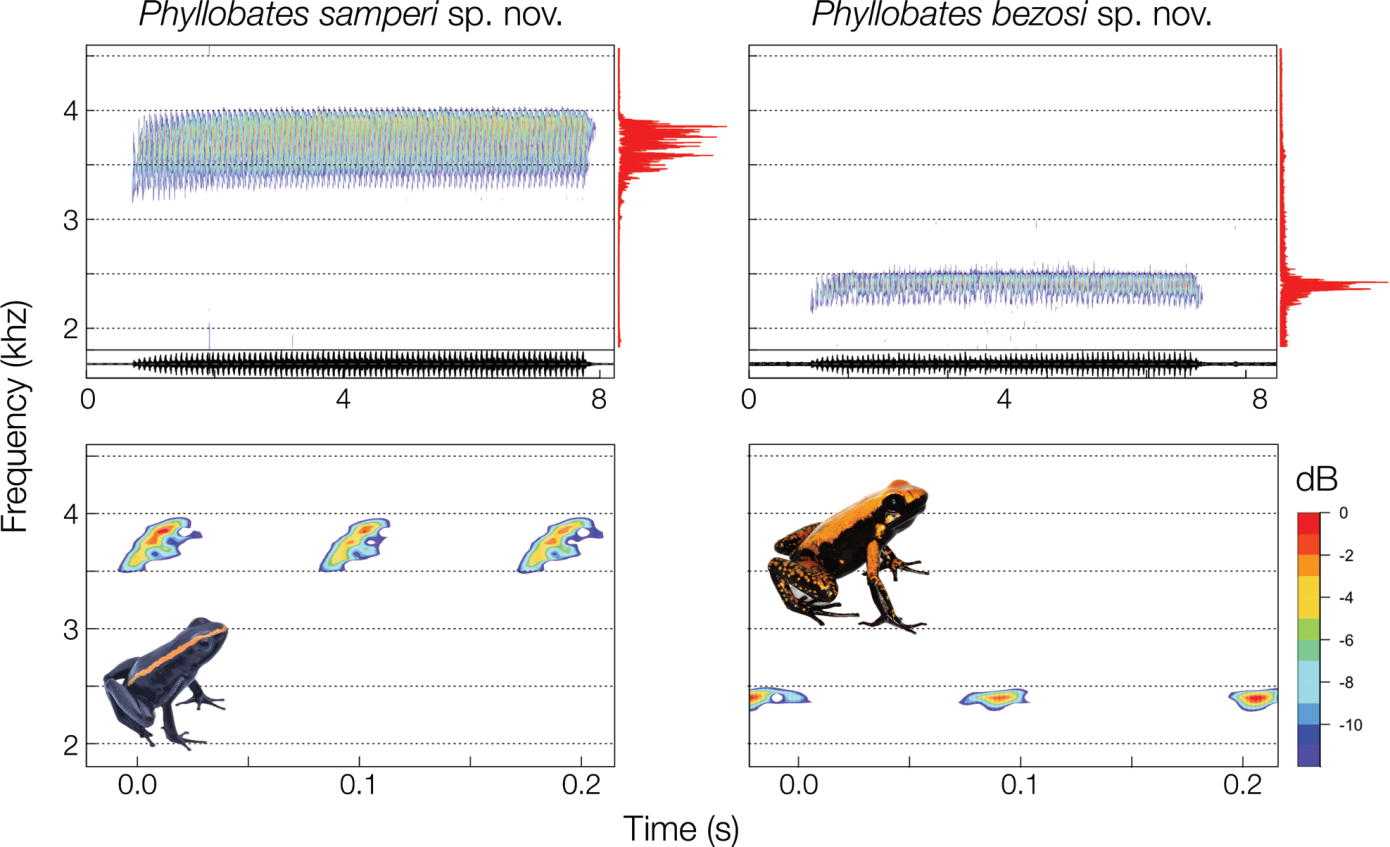
Advertisement calls. Sonagram of one series of advertisement calls (a trill, above) of the holotype male specimens of *Phyllobatessamperi* sp. nov. and *P.bezosi* sp. nov. An oscillogram (dark lines below the upper sonogram) shows a typical pattern of increase in sound pressure level. A power spectrum (red lines to the right of the upper sonograms) reveals most call energy is concentrated. In the sonograms below, a detail is presented of three uni-note calls per species.

The holotype is an adult male, 24.0 mm in snout-to-vent length, not dissected but assumed to be sexually mature because it was recorded (Fig. 5). In life it was pitch black with an orange dorsolateral stripe running from the tip of the snout to the posterior dorsum. All limbs black, dorsal and ventrally to the tip of fingers and toes. A few orange freckles were present in the dorsal surfaces of limbs and arms. Ventrally black. Body measurements are presented in Table 2.

##### Colouration.

The background colour of the head, back, sides, vent, and limbs is pitch black. A narrow golden yellow/orange dorsolateral stripe extends from the tip of the snout, along the outer margin of the upper eyelid, to the dorsum near the dorsal base of the thigh. In some individuals, a few tiny orange or greenish freckles are present on the dorsal surface of limbs and arms and near the dorsal surface of cloacae (Fig. 4). In preservative, the black background turns into a slightly greyish black, and the golden orange/yellow dorsolateral stripe and the specks become light grey to cream. Although the pattern remains discernible in specimens from 1985, the contrast between the dorsolateral stripe and the background seems to have faded, especially at the posterior end of the body.

##### Tadpole description.

**Figure 6.**
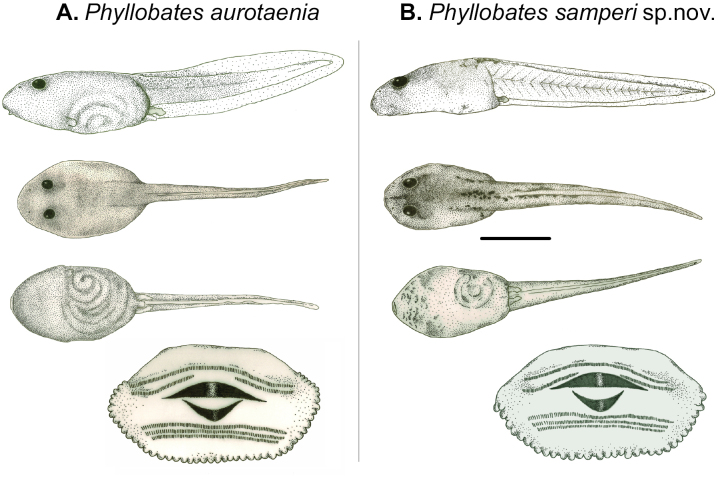
External morphology of the tadpoles of *Phyllobatessamperi* sp. nov. and, for comparison, with the previous putative species, *P.aurotaenia* in developmental stage 31 (Gosner 1960). Drawings by FV-S.

The following description is based on a single individual raised until stage 31 (Gosner 1960) from a clutch laid by a female in the laboratory (ANDES-A 3682; Fig. 6B). Body depressed, wider than high, ovoid in dorsal view; eyes and nostrils located dorsally. The spiracle is sinistral and the vent tube is dextral. Caudal musculature robust anteriorly and narrowing posteriorly; myotomic muscles are visible, tip of tail rounded, low dorsal fin, the ventral fin originates at vent tube. The oral disc is located anteroventrally with a 2/3 tooth formula; the second row of teeth above the mouth has a gap above the beak. The oral disc is anteriorly nude, the lateral and posterior edges of the disc have one row of papillae, and the beak is finely serrated. We collected an additional eight back-riding tadpoles at stage 25 from two of the paratypes (ANDES-A 3675 and 3676, four tadpoles each) from Magüipi, Buenaventura, Valle del Cauca. All nine tadpoles were deposited in the Herpetological collection at Universidad de los Andes (lots ANDES-A 3680, Andes-A 3681), and their measurements are presented in Table 3.

**Table 3.** Larvae. Developmental Gosner’s (1960) stage and body measurements of tadpoles of *P.samperi* sp. nov. collected during the current study.

ID	Stage	BL	TaL	ToL
ANDES-A 3682	31	8.04	13.98	22.02
ANDES-A 3680A	25	3.27	5.97	9.24
ANDES-A 3680B	25	3.41	6.1	9.59
ANDES-A 3680C	25	2.65	6.19	8.84
ANDES-A 3680D	25	3.16	6.87	10.03
ANDES-A 3681A	25	3.47	6.82	10.29
ANDES-A 3681B	25	4.1	6.69	10.79
ANDES-A 3681C	25	3.8	6.74	10.54
ANDES-A 3681D	25	3.68	6.68	10.36
Mean*		3.44	6.52	9.96
SD*		0.44	0.35	0.68

* Calculated only from stage 25 tadpoles.

##### Vocalisation.

*Phyllobatessamperi* sp. nov. utters trill calls. Each trill (Fig. 5) results from a long series of discrete sounds, produced through repeated thoracic compressions, one per sound. Because the number of sounds per trill strongly varies within and among individuals, a trill is best described as a call series rather than a multi-note call. Accordingly, males produce a series of 85.0 ± 16.7 (60–113, N = 10 males) uni-note calls. Series last 6.4 ± 1.3 s (4.7–8.8), individual calls 29 ± 5 ms (23–40), and intercall intervals 49 ± 6 ms (38–56). Call frequency peaks around 3.81 ± 0.08 kHz (3.72–3.95) and most (90%) energy is concentrated within a bandwidth of 0.44 ± 0.07 kHz (0.35–0.51). All recordings were made at body temperatures within 26.4–28.1 °C, a fairly narrow range that probably impeded the detection of statistical correlations between call traits and body temperature. Call duration of *P.samperi* sp. nov. (23–40 ms) overlaps with the range (23–30 ms) known for *P.aurotaenia* (González-Santoro et al. 2023), yet call peak frequency is generally higher (3.72–3.95 kHz) and barely overlaps with higher registered frequencies of the latter (3.27–3.77 kHz).

##### Ecology.

*Phyllobatessamperi* sp. nov. is a diurnal species that inhabits the understory of tropical humid forests in southwestern Colombia. From our observations, it thrives well in forests with moderate degrees of disturbance. Adults are found mainly on the leaf litter, near fallen logs, roots, large leaves, and other objects that provide refuge and probably food. Males defend territories by uttering advertisement calls usually from the leaf litter and less commonly from low perches; they sit near palm leaves, logs and roots, and often call while concealed under them. We observed very bold and aggressive responses when playing call recordings within males’ territories, including one individual hopping onto a researcher’s leg while looking for the invader. Males carry tadpoles on their backs and deposit them in ground-level phytotelmata, such as fallen palm bracts or leaf sheaths, or in semi-permanent puddles formed by rainwater on the forest floor. All of the collected back-riding tadpoles were at stage 25, matching the observation by Myers et al. (1978) for *P.terribilis*.

**Figure 7.**
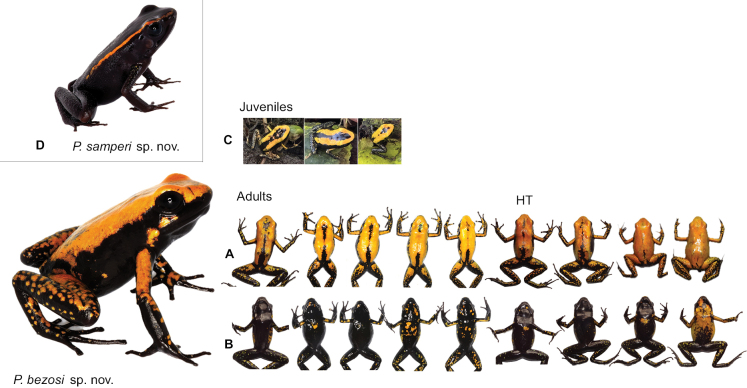
Colour variation among individuals of *Phyllobatesbezosi* sp. nov. (HT: holotype) including **A** dorsal and **B** ventral images for adult individuals and **C** three froglets, which exhibit the typical colouration with dorsolateral lines that characterises juvenile *Phyllobates*. To compare with juveniles of this species, we add a single image **D** of *P.samperi* sp. nov.

The colour pattern of *Pristimantisgaigeae* (Anura: Craugastoridae) closely resembles that of *Phyllobatessamperi* sp. nov. when found in sympatry. The same resemblance has also been reported for populations of *Pristimantisgaigeae* sympatric with *Phyllobateslugubris* and *Phyllobatesaurotaenia* (Myers and Daly, 1983). Since *Pristimantisgaigeae* is not known to be toxic, the resemblance may have resulted from Batesian mimicry, but the topic requires to be studied.

##### Distribution and remarks.

In the course of a parallel project on *Phyllobates*, one of the co-authors (P. Palacios-Rodríguez) visited the type locality of *P.aurotaenia* (Peñalisa, in the upper Condoto River) three times between 2014 and 2015. The site was severely degraded due to coca crops and gold mining and, despite intensive search of four people in three different years, no individuals of *Phyllobates* were seen or heard. We studied *P.aurotaenia* individuals from El Dos locality, 15 km north of the type locality, which exhibit the narrow greenish band that characterises *P.aurotaeniasensu lato* (this study; González-Santoro et al. 2023). The distance from the type locality to the closest study site is 12 km for *P.aurotaenia* and 140 km for *P.samperi* sp. nov.

**Figure 8.**
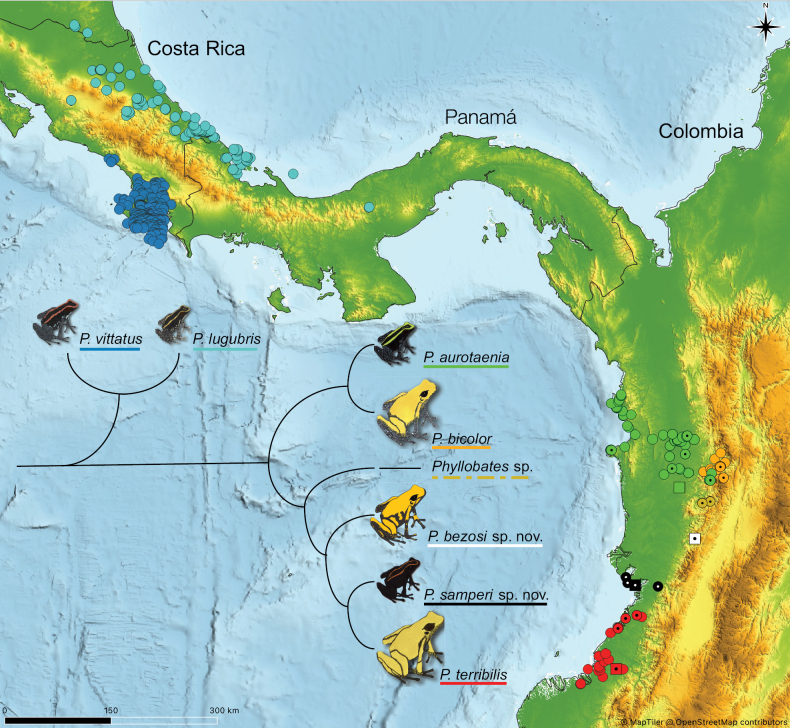
Systematics and estimated distribution of the seven *Phyllobates* species. Distribution was estimated from GBIF records (GBIF2023a–f), some of which were excluded based on well-acknowledged information (e.g., the genus distribution in South America is cis-Andean and not trans-Andean) as well as studies addressing distribution with the support of molecular data (Márquez et al. 2012; Márquez et al. 2020; González-Santoro et al. 2023). Dots with a point in the middle denote the localities in western Colombia that were sampled for molecular phylogenetic analyses in the current and previous studies (Márquez et al. 2020). Squares denote type localities for all species except *P.bicolor*. The tree topology follows the results depicted in Fig. 1. Frog icon colouration is approximate and should not be used to obtain diagnostic traits. See Fig. 4 for a colour diagnosis of the Colombian species.

To the best of our knowledge, the new species is distributed in the wet forests along the lower San Juan and Dagua river basins in the vicinities of Buenaventura, Valle del Cauca, Colombia, extending eastwards to the foothills of the Cordillera Occidental, but not surpassing elevations above 200 m a.s.l. (Fig. 8). To our knowledge, *Phyllobatessamperi* sp. nov. has not been reported south of the localities presented here. We suspect this species does not extend too far south of Buenaventura, since the lowland forests south of this municipality have been reasonably well sampled by herpetologists, including those working with *Phyllobates* (e.g. Silverstone 1975, 1976; Myers and Daly 1976; Lötters et al. 1997; Márquez et al. 2012, 2020). On the other hand, the northern limit to the distribution of *P.samperi* sp. nov. is unclear, since the medium and lower San Juan basin remain largely unexplored in terms of dendrobatid frogs. There are only two reports of *P.aurotaenia* south of Condoto, Chocó, and north of Buenaventura, both collected by Borys Malkin in Litoral del San Juan, Chocó, ~ 50–60 km north of Buenaventura, and deposited at the California Academy of Sciences (CAS HERP 119886 and 119445). As we did not examine these specimens, we refrain from modifying their taxonomic status.

##### Conservation.

Despite the uncertain northern limit of *P.samperi* sp. nov., the potential distribution range does not surpass 20,000 Km^2^. It is currently known from seven localities, all enclosed within a ~ 1000 km^2^ polygon, where deforestation due to agriculture, the expansion of urban Buenaventura, and gold mining operations will certainly reduce the amount and quality of habitat shortly. Based on these criteria, we propose listing *P.samperi* sp. nov. as Vulnerable (VU: B1a, biii, iv) under the IUCN Red List of Threatened Species, but this status should be re-evaluated as further information, especially on the species’s range, becomes available.

Uramba Bahía Málaga National Park is a marine reserve recently created by the Colombian Government to protect the marine, coastal and estuarine habitats of Bahía Málaga, just north of Buenaventura. Five of the localities presented in this study, including the type locality, are close to the protected coast of Bahía Málaga and should be, at least to some extent, protected by the presence of the reserve. Other fractions of the species’ range are within smaller forest reserves, such as the San Cipriano Forestal Protective Reserve, which allows for moderate extractive activities (e.g., logging). Most of this species’s range is, nevertheless, outside protected areas, and therefore still exposed to threats.

##### Etymology.

The species is named honouring Colombian biologist Cristian Samper, for his lasting impact on the world of conservation science and environmental stewardship. As founding director of the Alexander von Humboldt Biodiversity Institute of Colombia, President and CEO of the Wildlife Conservation Society, Director of the Smithsonian National Museum of Natural History and Managing Director of the Bezos Earth Fund, Samper is a leading voice in global conservation efforts, overseeing initiatives that span across 65 countries to protect 11.6 million square kilometres of wild places. Samper’s expertise in biodiversity and policy has been instrumental in shaping international dialogues around conservation, making him a respected figure in the intersection of science, governance, and activism.

#### ﻿ Phyllobates bezosi

Taxon classificationAnimaliaAnuraDendrobatidae

sp. nov.

C6FF6232-51E2-5E58-BEC4-225235B8CDA4

https://zoobank.org/1D579EC1-C66D-4441-A53A-E8BDBB094963

Figs 4 , 7

##### Type material.

***Holotype*** • Adult male. Field original label: AA_7256. Museum ID: CPZ-UV 9191. Type locality in Colombia, Valle del Cauca, municipality of Bolívar, valley of the Garrapatas river, 4.43°, -76.43°, 700 m elevation, riverine rainforest, amidst the leaf litter, July 2023. Collected by Wilmar Bolívar and Adolfo Amézquita. Advertisement calls of this male (Fig. 5) were recorded under laboratory conditions. Measurements of this individual are in Table 2. ***Paratopotypes*** • Three males and one female. Field original labels: AA_7257–60. Museum IDs: CPZ-UV 9192–5). All collected from the type locality, July 2023. Collected by Wilmar Bolívar and Adolfo Amézquita. Measurements of these individuals are presented in Table 2.

##### Diagnosis.

*Phyllobatesbezosi* sp. nov. is a medium-sized dendrobatid with an adult snout-to-vent length (SVL) of 31–36 mm (33.6 ± 1.7 mm, mean ± SD, *n* = 9). Body dorsal colouration is predominantly orange to yellow, with mid-dorsal black bands or blotches, usually incomplete and poorly defined, more frequently near the urostyle or between the eyes (Fig. 7). Laterally, the trunk shows suffusion of yellow into black. Dorsal surface of the limbs with irregular orange-to-yellow blotches on a predominantly black background. More yellow is generally found near the limb insertions. We also assign this species to *Phyllobates* based on other studies with more extensive outgroup sampling, including molecular (Santos et al. 2009) and combined (with morphological traits) phylogenetic analyses (Fig. 1; Grant et al. 2006, 2017; Márquez et al. 2020). We also use the following combination of traits: finger I longer than finger II, maxillary teeth present, and call of the trill type (Myers and Daly 1976; Myers et al. 1978).

##### Comparisons.

*Phyllobatesbezosi* sp. nov. can be externally distinguished from other yellow or orange species of its genus by the prevalence of black in the ventral colouration (Fig. 4). Both *P.terribilis* and *P.bicolor* exhibit predominantly yellow colouration ventrally, with some suffusion to black on the ventral surface of the hindlimbs and male throats. Despite large among-individual variations (Fig. 7), the ventral surfaces in *P.bezosi* sp. nov. are predominantly black in the throat, chest, belly, and limbs, with irregular orange to yellow blotches or sparks. There are no defined yellow blotches in *P.terribilis*, *P.bicolor* (Fig. 4), or its synonym *P.melanorrhinus* (Myers & Böhme, 1996): none of the six specimens of the latter exhibit evidence of well-defined colour blotches in dorsal or ventral colouration, and dorsal colouration remains light grey in preservative (Myers and Böhme 1996: figs 1–3), whereas it turns into dark black in *P.bezosi* sp. nov. The flanks are also predominantly black in nearly all individuals of *P.bezosi* sp. nov. but yellow in the other two species. The froglets of *P.bezosi* sp. nov. also exhibit bright dorsolateral stripes shortly after metamorphosis (Fig. 7), but the stripe changes to dominant orange-to-yellow dorsal colouration during development. The absence of dorsolateral stripes in adults also distinguishes *P.bezosi* sp. nov. from all forms of *P.aurotaenia*, *P.samperi* sp. nov. (Figs 4, 7), as well as the Central American species *P.lugubris* and *P.vittatus*.

##### Description of the holotype.

The holotype is an adult male, 32.6 mm in snout-to-vent length, not dissected but assumed to be sexually mature because it was observed calling and recorded in captivity (Fig. 4). In life it was predominantly orange in the dorsum with middorsal discontinuous black bands of variable width (Figs 4, 7). Rostrum orange dorsally and black ventrally, with the two colours present in the upper jaw and without any colour suffusion. Lateral body black, without any suffusion to the orange dorsum, with a few orange blotches. Groins black. Dorsal surface of arms orange, and of forearms black with orange blotches. Dorsal surface of legs black with orange blotches. Ventral surface of all limbs predominantly black with a few orange blotches in the arms and yellow to greenish blotches in the legs. Ventrally black with a few rounded yellow blotches, variable in size. Body measurements are presented in Table 2.

##### Colouration.

Adult individuals of *P.bezosi* sp. nov. are predominantly orange to yellow in the dorsum, more often with mid-dorsal black bands or blotches that are incomplete. The orange/yellow is more often replaced by predominantly black on the flanks, limbs, and ventral surfaces. A few individuals do not bear the middorsal black bands (Fig. 7) and yellow can extend towards the flanks and venter, but never with blue blotches or speckles, as in *P.bicolor*. Ventrally, adults can be completely pitch black, with a single golden blotch near the chest, or with a variable number and size of golden blotches. In preservative (70% ethanol), the individuals look completely black. Some remain dark grey in the areas previously covered by yellow colouration. All ventral yellow or golden blotches or speckles fade out.

##### Vocalisation.

*Phyllobatesbezosi* sp. nov. males utter trill calls (Fig. 5). Males produce a series of 95 (76–113, *n* = 2 males) uni-note calls. Series last 9.5 s (7.8–11.1), individual calls 36 ms (37–38), and intercall intervals 64 ms (61–66). In contrast to *P.samperi* sp. nov., the call frequency peaks around 2.4 kHz (2.38–2.43). Interestingly, most (90%) energy is concentrated within a much narrower bandwidth of 0.30 kHz (0.27–0.34), but the low number of recorded males (2) does not allow us to elaborate on this topic. The two recordings were made at body temperatures of 21.3 and 21.7 °C, respectively. Call duration of *P.bezosi* sp. nov. (37–38 ms) overlaps with the range (35–39 ms) known for *P.bicolor* (González-Santoro et al. 2022, 2023). The peak frequency in the two recorded individuals of the former (2.38–2.43 kHz) falls also well within the range of frequencies of the latter (1.99–3.07 kHz).

##### Ecology.

During its time in the laboratory, a couple bred. A male was found carrying nine tadpoles on his back (7 June 2023). After placing him in another container with water, tadpoles were found free within 24 h (June 8). They were kept in a 25 × 20 × 20 cm aquarium, with algae, aquatic vegetation and supplemented with fish food. The first metamorph was found after 74 days (21 August) and the last one after 94 days (September 10). They expressed very early the two dorsolateral yellow bands that characterise the colouration of *Phyllobates* juveniles (Fig. 7). Other aspects of territoriality, habitat use, and breeding are likely to be similar to the other *Phyllobates* species, but our time in the field was too short to corroborate this.

##### Distribution and conservation.

The species is known so far from a single locality: the type locality at the Garrapatas River (municipality of Bolívar, Valle del Cauca). The information on the status of the forests is very poor. Nonetheless, this area has suffered strong and increasing violence during the last decade, mostly associated with intensive illegal gold mining and narcotraffic. At the moment of writing this manuscript, access to the area is restricted by illegally armed groups that also constrain the movement of local inhabitants. Besides the aforementioned illegal activities, the lack of governmental authorities in the area and the long history of dendrobatid poaching in Valle del Cauca and Chocó allow us to infer a high risk for the species described herein. We thus propose to declare it Endangered EN B1ab(iii), B2ac(iii), under the IUCN Red List criteria (IUCN 2012), until additional information is collected.

##### Etymology.

The species is named honouring American entrepreneur Jeff Bezos, for his contributions to environmental conservation, particularly through the Bezos Earth Fund. The fund aims to combat climate change and preserve the natural world by funding scientists, NGOs, and other local communities dedicated to environmental action. The fund’s investments in conservation, restoration initiatives, food systems, clean energy, and sustainable development reflect a commitment to stewarding the planet for future generations. The considerable financial resources allocated for environmental causes symbolise an important step in mobilising private capital for public good, particularly in an era urgently demanding climate solutions. The name of this new remarkable species is expected to highlight this hallmark decision.

## ﻿Discussion

The Colombian Pacific lowlands are under strong pressure by the growth of illegal activities. Illegal mining destroys the riverine forests and riverbeds at unprecedented rates, and contaminates the water. Besides, the co-occurrence of gold mining with illegal crops favours the settlement and control of activities and routes by illegally armed groups. One of these activities is the poaching of dendrobatid frogs. Under this scenario, scientific and political decisions are badly needed to improve the survival chances of organisms therein. The study and acknowledgement of evolutionary unique lineages should decisively contribute to the quality of decisions in conservation.

To improve the coherence between previous molecular studies (Márquez et al. 2020) and current taxonomy, this study adds a series of specimens and formally proposes a new taxonomic arrangement for the Colombian *Phyllobates* (Fig. 8). This research increases the number of three to six species, one of which (*P.bicolor* South in Márquez et al. 2020; *Phyllobates* sp., this study) has not been described yet. As explained above, the security situation in its known distribution area precludes further sampling to formally describe it.

The three species of the southern Colombian clade now include two lowland and latitudinally separated species (Fig. 8), *P.terribilis* and *P.samperi* sp. nov., and two species that seem to thrive at slightly higher altitudes, *P.bezosi* sp. nov. and *Phyllobates* sp., although our knowledge on the distribution of the latter two remains limited. The three named clades are monophyletic, easily diagnosable from external colouration, and genetically distant enough to be named and protected as evolutionarily independent lineages.

The taxonomic uncertainty is higher for the remaining populations of *P.aurotaenia* that together with *P.bicolor* form the northern clade of Colombian *Phyllobates*. Recent molecular (Márquez et al. 2020) and bioacoustic (González-Santoro et al. 2023) evidence are compatible with the existence of introgression between the two species, which would have at least partly led to observed colour variation in *P.aurotaenia*. Myers et al. (1978) had first speculated that this morph could be the product of introgressive hybridization between *P.aurotaenia* and *P.bicolor*. Introgression would also explain Silverstone’s (1976) notion that yellow-banded (a.k.a. broad-banded) *P.aurotaenia* represents a distinct form, originally based on their wider dorsolateral bands and larger size. Further work is needed to resolve this intriguing issue, including additional morphological, morphometric, acoustic, and multi-locus genotypic data from more populations of the respective taxa. This research is needed to feed back into political decisions on the conservation of those lineages.

Finally, the phylogenetic hypothesis and taxonomic arrangement presented here corroborate an interesting pattern observed in previous studies (Grant et al. 2006; Santos et al. 2009; Márquez et al. 2012, 2020; González-Santoro et al. 2023). The fact that pairs of predominantly yellow/orange species, *P.terribilis* – *P.bicolor* and *P.terribilis* – *P.bezosi* sp. nov., do not group as sister species supports that solid-yellow dorsal colouration, along with increased toxicity and body size, evolved in at least two independent events along the phylogenetic history of *Phyllobates* (Márquez et al. 2020). Since these traits are considered part of an aposematic syndrome in dendrobatid frogs (Summers and Clough 2001; Santos and Cannatella 2011) the genus offers replicated evolution of correlated traits, a valuable research system to study the mechanisms promoting the evolution of conspicuous warning colouration.

## Supplementary Material

XML Treatment for Phyllobates samperi

XML Treatment for Phyllobates bezosi

## ﻿Additional information

### Author ORCIDs

Adolfo Amézquita https://orcid.org/0000-0001-7320-1143

Michelle Quiroz https://orcid.org/0000-0002-6955-4605

Luis A. Mazariegos-H https://orcid.org/0000-0003-4882-0414

### Data availability

All of the data that support the findings of this study are available in the main text or Supplementary Information.

## Supplementary materials

Supplementary material 1 Linked data for primary biodiversity data Data type xlsx https://binary.pensoft.net/file/1135009This dataset is made available under the Open Database License (http://opendatacommons.org/licenses/odbl/1.0/). The Open Database License (ODbL) is a license agreement intended to allow users to freely share, modify, and use this Dataset while maintaining this same freedom for others, provided that the original source and author(s) are credited.Adolfo Amézquita, Fernando Vargas-Salinas, Iván Ramos, Pablo Palacios-Rodríguez, Erika Nathalia Salazar, Michelle Quiroz, Wilmar Bolívar, Diana M. Galindo-Uribe, Luis A. Mazariegos-H
